# Fused tracks and layers of Ti10Mo6Cu data obtained via laser powder bed fusion

**DOI:** 10.1016/j.dib.2022.108775

**Published:** 2022-11-23

**Authors:** Thywill Cephas Dzogbewu, Willie Bouwer du Preez

**Affiliations:** a Department of Mechanical and Mechatronics Engineering, Faculty of Engineering, Built Environment and Information Technology, Central University of Technology, Free State, Bloemfontein, South Africa; b Centre for Rapid Prototyping and Manufacturing, Faculty of Engineering, Built Environment and Information Technology, Central University of Technology, Free State, Bloemfontein, South Africa

**Keywords:** Fused tracks analysis, Double layers analysis, Laser powder bed fusion, Ti10Mo6Cu

## Abstract

Laser powder bed fusion (LPBF) has opened the window of in-situ alloying elemental powders for specific engineering and biomedical applications. However, since the LPBF process is non-linear, and the current numerical models are still at the experimental stage it is obligatory to determine the optimum process parameters for each powder composition. The current experimental data described the effects of laser powers and scanning speeds on fused tracks and layers produced using Ti10Mo6Cu powder blend. Fused single tracks were produced at varying scanning speeds and laser powers. The process parameter that falls within the conduction mode threshold was used to produce double layers at varied hatch distances. Layers were rescanned at an offset distance of half the hatch distances. The fused tracks and layers were metallurgically prepared according to the Struers protocol and etched with Kroll's reagent. Optical and scanning electron microscopes were used to measure the width (W), depth of penetration (D), and height (H) of the fused tracks to obtain the data for characterizing the geometry of the fused tracks. Data on the surface quality of the fused layers were extracted with a Surftest SJ-210 portable surface roughness tester, while microhardness test data was extracted using a FM-700 Digital Vickers Microhardness Tester. The data obtained could be used for validating numerical and analytical models, and for predicting fused track profiles. Data that originated from the layers could be used to predict the morphology of layers and the dispersion of elements during in-situ alloying. The methodology applied could be used by other researchers to determine the process parameters for other powder blend compositions and increase the materials database for the LPBF process.

**Specifications Table**

**Table:** 

Subject	Industrial and Manufacturing Engineering
Specific subject area	Additive Manufacturing, Laser Powder Bed Fusion, Selective Laser Melting
Type of data	Tables, Images, Charts, Graphs, Figures
How the data were acquired	Scanning electron microscope (SEM), Optical Microscope (OM), Surface roughness measurement machine, Microhardness measurement device
Data format	Raw and Analyzed
Description of data collection	An EOS M280 machine was used to produce fused tracks and double layers. Data on the width (W), penetration depth (D), and height (H) of the fused tracks were collected to determine the effects of varied laser power and scanning speed on the geometry of the fused tracks. Data was collected on the morphology and elemental dispersion from the double layers. Surface roughness of the layers were measured to extract data on the quality of the layers. Cross-section micro-hardness was measured at different inspection points of the fused tracks and layers.
Data source location	Institution: Department of Mechanical and Mechatronics Engineering and Centre for Rapid Prototyping and Manufacturing, Faculty of Engineering, Built Environment and Information Technology, Central University of Technology, Free State, Bloemfontein 9300, South African.
Data accessibility	Data is available within this article and at the Mendeley Repository at https://data.mendeley.com/datasets/fcs8nfwmpc/1

## Value of the Data

• The data extracted from the experimental investigation could be used to validate numerical and analytical models for laser powder bed fusion processes.
• The data from the morphology and geometry of the fused tracks could be used to develop and validate a novel approach for the prediction of various track profiles (keyhole mode and conduction mode profiles) and other abnormalizes (balling effect, spattering, humping effect, etc.).
• The data could serve as a guide for other researchers to predict the window of process parameters that could yield optimum process parameters for TixMoxCu powder compositions.
• The data obtained from the morphology and EDS (energy dispersive spectroscopy) elemental mapping could be used to predict the surface quality and elemental homogeneity of LPBF built components based on the selected hatch distance.
• Most previous studies based their process parameter optimization analysis on only one fused track per process parameter (laser power and scanning speed). Such an approach does not prove the repeatability of the process. The current analysis is based on three fused tracks under the same laser melting conditions that give evidence of the repeatability of an optimum process parameter.
• Laser powder bed fusion researchers in industries and academia could benefit from the data for various research activities.

## 1 Objective

The objective of generating the data was to understand the effect of principle process parameters (laser power, scanning speed, hatch distance) on fused single tracks and layers by in-situ alloying elemental powders (Ti, Mo, Cu) via the LPBF process. It is very important to understand how the laser beam interacts with the powder bed. Understanding such a phenomenon helps in determining the optimum process parameters for each composition of elemental powders for various applications. Understanding the laser-matter interaction of fused single tracks and layers is paramount in understanding the layer-wise process of building 3D structures using the LPBF process.

## 2 Data Description

### 2.1 Data summary

**Fig. 2:**
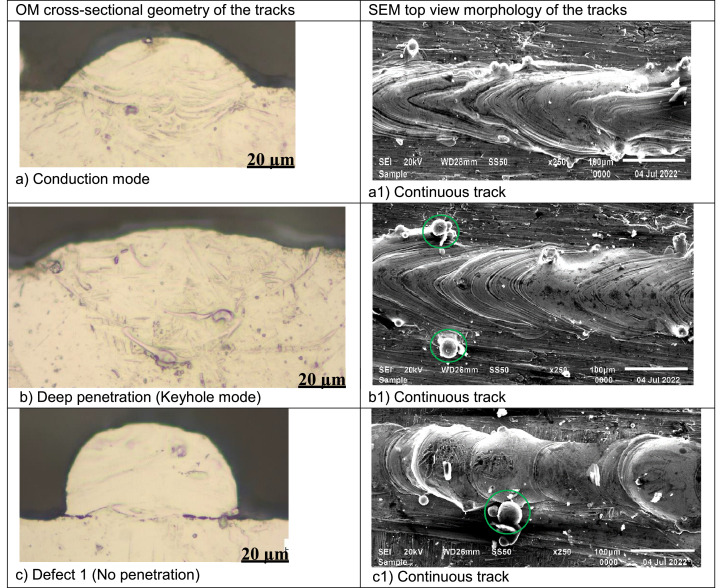

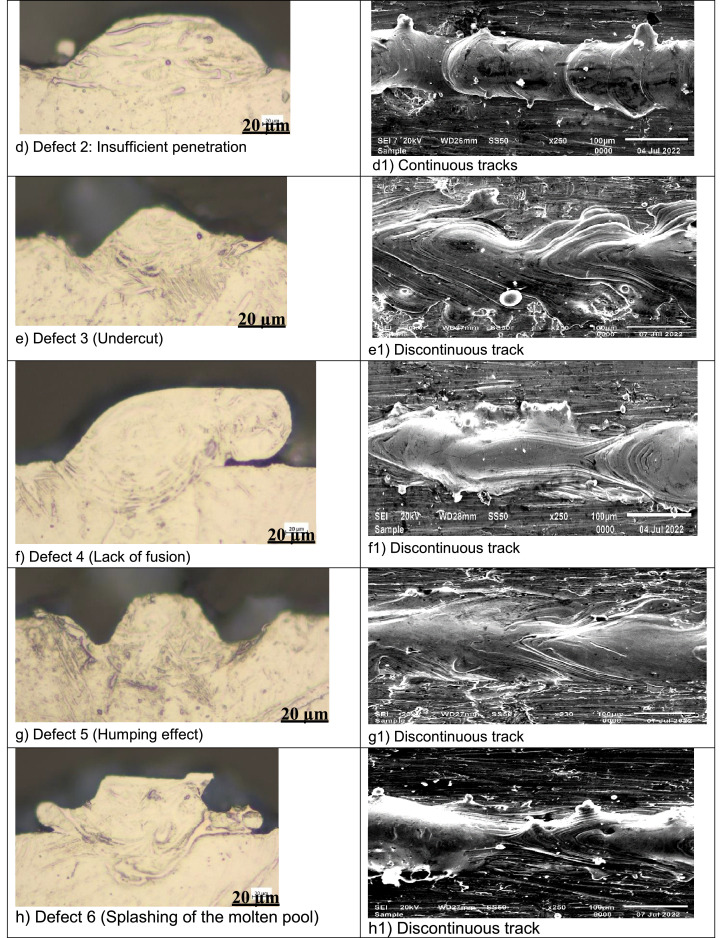
Geometry and morphology of the fused tracks at various process parameters, (a) Conduction mode: 150 W, 1.5 m/s; (b) Deep penetration: 150 W, 0.8 m/s; (c) No penetration: 50 W, 0.4 m/s; (d) Insufficient penetration: 50 W, 0.1 m/s; (e) Undercut: 300 W, 1.8 m/s; (f) Lack of fusion: 200 W, 2.2 m/s; (g) Humping effect: 300 W, 2.2 m/s; (h) Splashing of the molten pool: 150 W, 1.6 m/s (a1) Continuous track: 150 W, 1.5 m/s; (b1) Continuous track with satellites – green circle: 150 W, 0.8 m/s; (c1) Continuous track with satellites – green circle: 50 W, 0.4 m/s; (d1) continuous track: 150 W, 0.1 m/s (e1) Discontinuous track: 300 W, 2.2 m/s (f1) Discontinuous track: 200 W, 2.2 m/s; (g1) Discontinuous track: 100 W, 1.0 m/s;(h1) Discontinuous track: 150 W, 1.6 m/s.
• Deep penetration (keyhole mode): The laser energy was able to melt the composite powder, produce continuous tracks, and ‘drill’ into the substrate (Fig. 2b) [12].
• No penetration - The laser energy could melt the composite powder, and form continuous tracks from the top view, but could not bind metallurgically with the substrate (the previous layer) from the cross-sectional view (Fig. 2c) [13].
• Shallow penetration - The laser energy could melt the composite powder, and form continuous tracks from the top view but could not penetrate the substrate sufficiently as observed from the cross-sectional view (Fig. 2d) [14].
• Defects (undercut, lack of fusion, humping effect, splashing of the molten pool): The laser energy could melt the constituent powder, form varied discontinuous tracks from the top view and imperfect track geometry from the cross-sectional view (Fig. 2e-h) [13,15].

For the LPBF process to produce 3D structures with accurate geometrical characteristics of each powder composition, it is obligatory to determine the process parameters that could be used for each powder constituent [1,2]. Titanium and its alloys have been used extensively for many strategic applications in the biomedical, energy, automobile and aerospace industries [3], [4], [5]. The development of Ti-based alloys and the determination of process parameters to produce Ti-based alloys via LPBF would certainly broaden the material database for the additive manufacturing process. The LPBF process would also enable the manufacturing of near-net shape parts [6,7]. Therefore, the data presented in this paper is of crucial importance, since the research was focused on optimizing process parameters that could be used to produce a Ti-based alloy (Ti10Mo6Cu) for specific industrial applications. The data was collected according to detailed experimental design procedures [8,9]. The main design factors were laser power, scanning speed, and hatch distance. A detailed description of the data is presented below:

• Selected manufacturing process conditions (Fig. 7) and raw material data such as powder characterization (Fig. 6) are presented below.
• Data on the morphological and geometrical characterization of the fused tracks and layers can be found at the Mendeley link reported above and codified according to the file format ”P_v.jpg”, where P is the laser power in W and v is the scanning speed in m/s.
• The measurement of the fused track geometry obtained from the microscopic analysis of the top and cross-sectional view (Fig. 2) is provided in a spreadsheet that can be found at the Mendeley link reported above. From the top view of the fussed tracks, at least 10 measurements were taken along the width of the tracks. The averages of the readings were plotted (Fig. 3) against the laser power and scanning speed.
• The surface roughness of the double layers was measured and captured on a spreadsheet that can be found at the Mendeley link reported above. At least nine measurements were taken. The average of the measurements at the varied hatch distances were plotted (Fig. 4c)
• The microhardness of the fused tracks and layers was measured and plotted (Fig. 5). At least twenty indentations were made for each sample and the average values were plotted. The raw data can be found at the Mendeley link provided above.

### 2.2 Morphology of the fused tracks

**Fig. 1:**
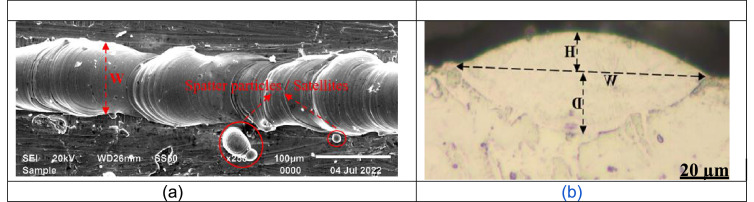
(a) SEM top view morphology of fused tracks (b) OM cross-sectional geometrical characteristics of the fused tracks.

The data on the morphology of the fused tracks was collected using a scanning electron microscope (Fig. 1a). The fused tracks were cross-section and metallurgically prepared according to Struers protocol [10]. The geometry of the fused tracks was measured (Fig. 1b). Based on the morphology and geometrical characteristics the fused tracks were classified into five distinct groups, as follows:

• Conduction mode: The laser energy was able to melt the composite powder, form continuous tracks, and penetrate the substrate forming a U shape profile (Fig. 2a) [11].

### 2.3 Geometrical characterization of the fused tracks

**Fig. 3:**
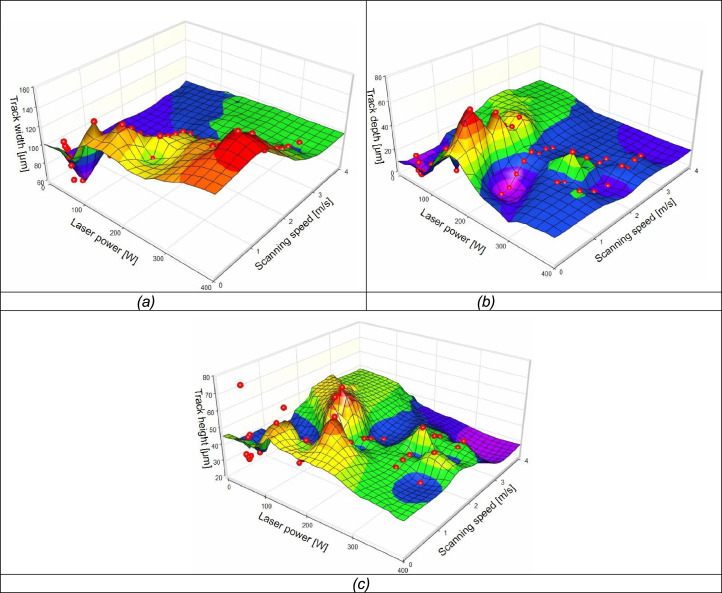
Geometrical characteristic relationships between fused tracks at different laser powers and scanning speeds: (a) Track width, (b) Track depth, (c) Track height.

The geometrical characteristics of the fused tracks were measured (Fig. 1b). The statistical representation in Fig. 3 reveals the relationship between the geometrical characteristics of the fused tracks at the various laser powers and scanning speeds.

### 2.4 Morphology, geometrical characterization, and microhardness data of the double layers

Double layers were produced using the process parameters obtained for conduction mode (Fig. 2a). The layers were produced at varied hatch distances of 80 µm, 90 µm, and 100 µm, under two exposures (single scan and rescan – Table 1). The following data was extracted

**Fig. 4:**
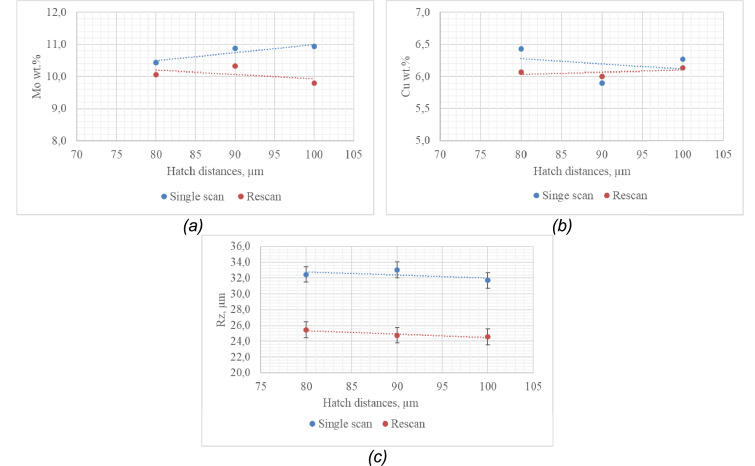
(a) Elemental concentration of Mo in the Ti10Mo6Cu alloy matrix, (b) Elemental concentration of Cu in the Ti10Mo6Cu alloy matrix, (c)Surface roughness of the layers, all as function of the hatch distance.
• The surface roughness of the samples varied at the various hatch distances (Fig. 4c)
• The elemental compositions of the rescan samples were different from the elemental composition of the single scan samples (Fig. 4a-b).
• There were pockets of elemental concentration of the constituent elements in the Ti10Mo6Cu matrix (Table 1- the black circles on the EDS maps).
• The microhardness values of the fused tracks and layers were different (Fig. 5)

• There were satellites on the surfaces of the samples (Table 1 – single scan, red circles)
• The surface roughness of the single scan samples was different from the surface roughness of the rescan samples (Table 1 and Fig. 4c).

**Fig. 5:**
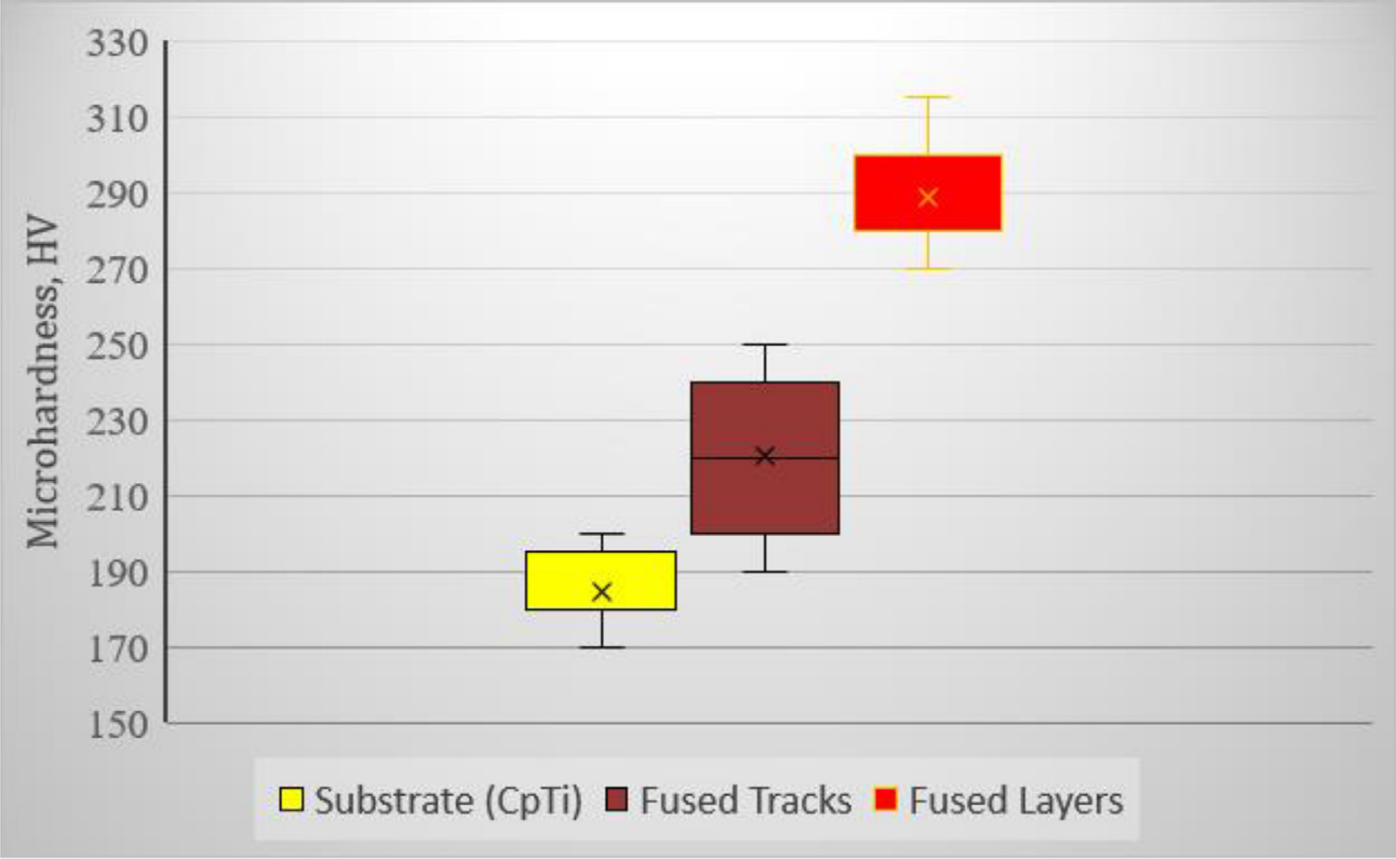
Microhardness data of the Ti10Mo6Cu fused tracks and layers.

**Table 1:** Morphology and EDS elemental mapping of the fused layers (red circles – satellites, black circles - elemental concentration)

### 2.5 Microhardness

The box plot in Fig. 5 presents the data extracted from the microhardness tests on the substrate, fused tracks and fused layers.

## 3 Experimental Design, Materials, and Methods

The samples were produced using an Electro-Optical Systems GmbH (EOS) M280 machine equipped with a continuous-wave ytterbium fibre laser operating at 400 watt. The laser beam had a TEM00 Gaussian profile of 80 µm spot size and argon gas was used as the protective atmosphere (inert gas). The main components of the machine are a process chamber, an automatic powder delivery system, a building platform, a process gas management system and a computerized system for process control.

**Fig. 6:**
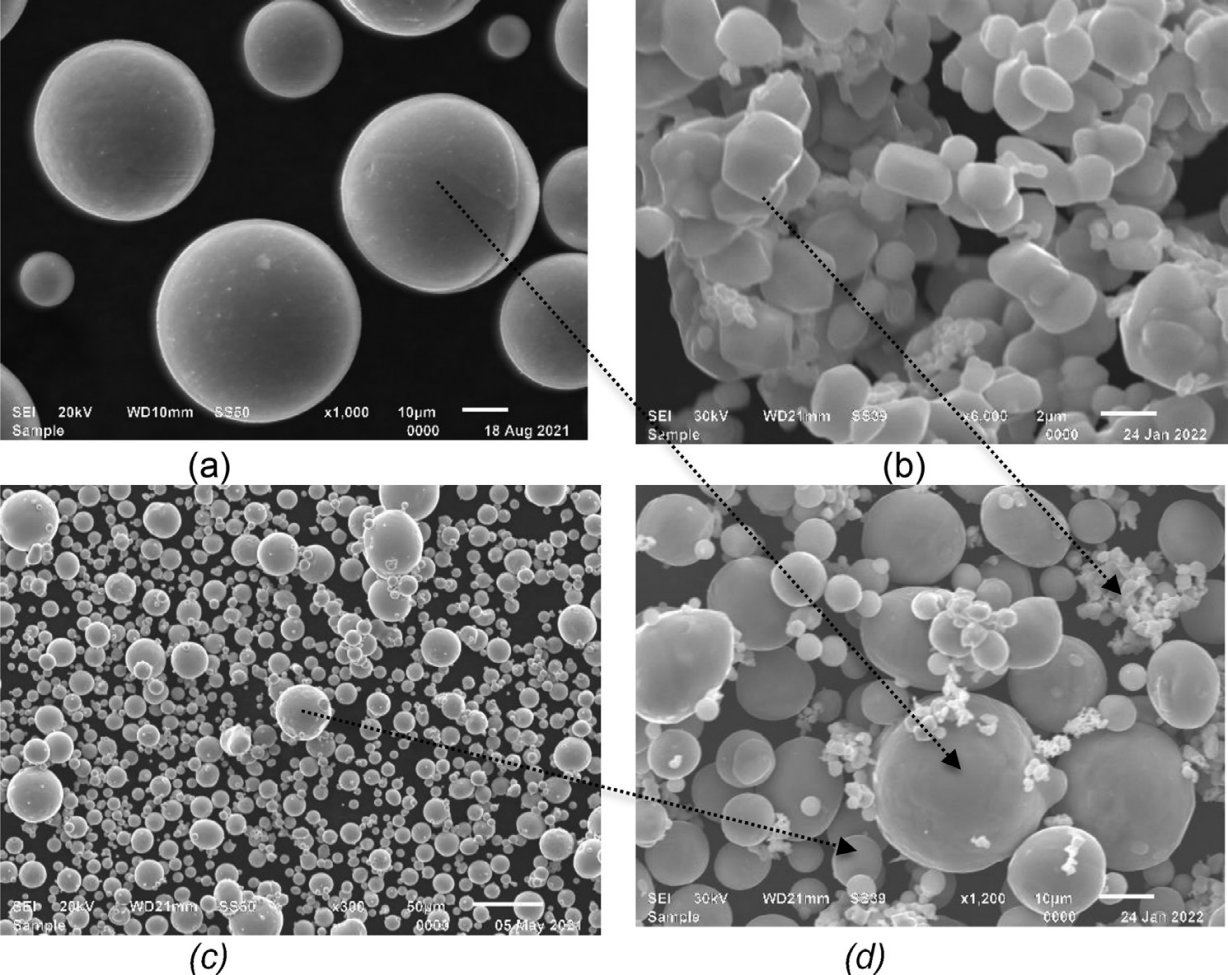
SEM images of the powders: a) CP Ti powder, b) Mo powder, c) Cu powder, d) Powder blend (Ti10Mo6Cu).

The samples were produced using gas-atomized Ti (Cp Ti, grade 2) powder, Mo, and Cu powders which were supplied by Sabinano (Pty) Ltd, Johannesburg, South Africa. The nominal chemical composition of the Cp Ti powder as received was: Ti (bal.), O (0.17), Fe (0.062), C (0.006), H (0.002) and N (0.012) in weight percent (wt.%). The Mo and Cu powder was of 99.9 % purity. The particle size distribution of the Cp Ti in sieve diameters weight by volume were: d10 = 12.6 μm, d50 = 29.8 μm and d90 = 41.5 μm and that of Cu were d10 = 9.45 μm, d50 = 21.9 μm and d90 = 37.5 μm. Cp Ti powder and Cu powder particles were highly spherical with smooth surfaces (Fig. 6a & b). The Mo powder had a plate-like shape (Fig. 6c) and an average diameter of 1 µm with a smooth surface. The feedstock for the production of the samples was obtained by mechanically mixing 84 wt.% of Ti, 10 wt.% of Mo and 6 wt.% of Cu which is referred to as Ti10Mo6Cu (Fig. 6d). A 3D Turbula-mixer procured from SSP Processing Equipment (Pty) Ltd, Johannesburg, South Africa was used to mix the powders for 30 min at 150 rpm.

**Fig. 7:**
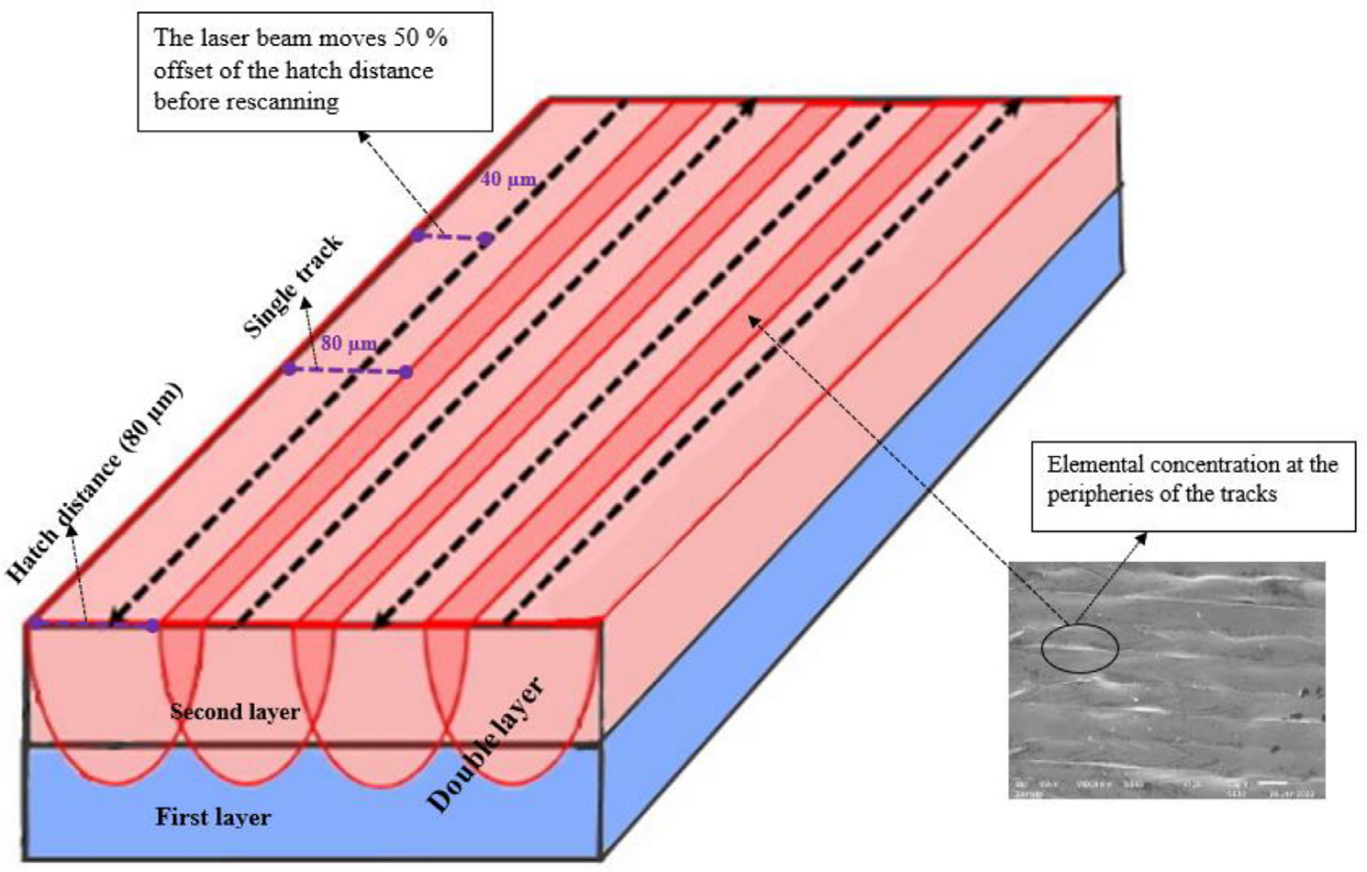
A schematic representation of the rescanning strategy.

Fused tracks were produced over a wide range of scanning speeds (0.08–3.4 m/s) and laser powers (50–350 W) on a Cp Ti substrate with a chemical composition similar to that of the Cp Ti precursor powder. The powder layer thickness was 50 µm. Three fused tracks were produced for each combination of process parameters for statistical analysis and proving the repeatability of the process parameters. The length of the tracks was 10 mm with 1 mm between neighbouring tracks. The width of the tracks from the top view was measured (Fig. 1a). After measuring the width of the fused tracks, the tracks were cross-sectioned and metallurgically prepared according to Struers metallurgical preparation protocol [10] (grinding with 320 papers, polishing by diamond suspensions (9, 3, 1 µm size) and etched with Kroll's reagent. The geometry of the fused tracks was characterized by measuring the width (W), height (H), and penetration depth (D) (Fig. 1b). The process parameter set that exhibited a conduction mode profile (Fig. 2a) was used to produce double layers at varied hatch distances of 80 µm, 90 µm and 100 µm for the determination of the optimum hatch distance for subsequent manufacturing the Ti10Mo6Cu samples. The double layers were produced at hatch distances of 80 µm, 90 µm and 100 µm and rescanned at a 50% offset hatch distance (Thus, for the 80 µm hatch distance, the laser moved 40 µm before rescanning, and for 90 µm and 100 µm, it moved 45 µm and 50 µm, respectively, before rescanning) (Fig. 7).

The data on the morphology and the distribution of Mo and Cu in the Ti10Mo6Cu alloy matrix at various hatch distances was extracted using optical and scanning electron microscopy (Table 1 and Fig. 4 a-b). The surface roughness of the fused layers was measured using a Surftest SJ-210 portable surface roughness tester procured from Mitutoyo America Corporation (Fig. 4c). Microhardness tests of the samples were conducted on polished cross-sections perpendicular to the building direction at 200 g loading for a holding time of 15 s. (20 measurements were taken for each cross-section for statistical purposes) (Fig. 5).

## Ethics Statements

Not applicable.

## CRediT authorship contribution statement

**Thywill Cephas Dzogbewu:** Conceptualization, Methodology, Software, Data curation, Writing – original draft. **Willie Bouwer du Preez:** Visualization, Investigation, Supervision, Software, Validation, Writing – review & editing.

## Declaration of Competing Interest

The authors declare that they have no known competing financial interests or personal relationships that could have appeared to influence the work reported in this paper.

## Data Availability

Fused tracks and layers of Ti10Mo6Cu data obtained via laser powder bed fusion (Original data) (Mendeley Data)
